# Antimicrobial Efficiency of *Aloe arborescens* and *Aloe barbadensis* Natural and Commercial Products

**DOI:** 10.3390/plants10010092

**Published:** 2021-01-05

**Authors:** Kaja Kupnik, Mateja Primožič, Željko Knez, Maja Leitgeb

**Affiliations:** 1 Laboratory for Separation Processes and Product Design, Faculty of Chemistry and Chemical Engineering, University of Maribor, Smetanova Ulica 17, 2000 Maribor, Slovenia; kaja.kupnik@um.si (K.K.); mateja.primozic@um.si (M.P.); zeljko.knez@um.si (Ž.K.); 2 Faculty of Mechanical Engineering, University of Maribor, Smetanova Ulica 17, 2000 Maribor, Slovenia; 3 Faculty of Medicine, University of Maribor, Taborska Ulica 8, 2000 Maribor, Slovenia

**Keywords:** aloe vera, antimicrobial activity, natural aloe products, commercial aloe products

## Abstract

Nowadays, there are many commercial products from natural resources on the market, but they still have many additives to increase their biological activities. On the other hand, there is particular interest in natural sources that would have antimicrobial properties themselves and would inhibit the growth and the reproduction of opportunistic microorganisms. Therefore, a comparative antimicrobial study of natural samples of aloe and its commercial products was performed. Qualitative and quantitative determination of antimicrobial efficiency of *Aloe arborescens* and *Aloe barbadensis* and its commercial products on fungi, Gram-negative, and Gram-positive bacteria was performed. Samples exhibited antimicrobial activity and slowed down the growth of all tested microorganisms. Research has shown that natural juices and gels of *A. arborescens* and *A. barbadensis* are at higher added concentrations comparable to commercial aloe products, especially against microbial cultures of *Bacillus cereus*, *Candida albicans*, and *Pseudomonas aeruginosa*, whose growths were completely inhibited at a microbial concentration of 600 μg/mL. Of particular importance are the findings of the good antimicrobial efficacy of fresh juice and gel of *A. arborescens* on tested microorganisms, which is less known and less researched. These results show great potential of *A. arborescens* for further use in medicine, cosmetics, food, and pharmaceutical industries.

## 1. Introduction

Various natural raw materials from plants (fruits, herbs, seeds, spices, and vegetables), animals (eggs, milk, tissues, and mucus), microorganisms (bacteria and fungi), and their extracts are interesting, as they exhibit various pharmaceutical, medicinal, and other biological activities [1,2,3]. One of the most applied herbal medicines worldwide is *A. barbadensis*, a succulent plant with many beneficial properties. It was considered as a blessing to mankind by different ancient physicians. Even the various names such as “magic plant”, “wonder plant”, and “nature healer” as well as the fact that it is one of the oldest mentioned plants with healing properties and health benefits say a lot about how popular it is in various branches of traditional medicine such as Ayurveda, siddha, homeopathy, and Unani [4,5].

In addition to the well-known *A. barbadensis*, there are many different species of *Aloaceae* family [6]. One of them is *A. arborescens*, which is said to contain even more active ingredients than *A. barbadensis* [7]. Furthermore, the most commercially used among all species of the genus *Aloe* is *A. barbadensis*, mainly due to the gel content being higher compared to other species [8]. In recent years, the industrial use of aloe vera has developed greatly, as the gel and the juice are used in the food as well as the pharmaceutical and the cosmetic industries [9]. In cosmetics, the gel is often used as a base for creams, lotions, soaps, shampoos, facial cleansing tonics, and other products due to its moisturizing effect [10]. In the pharmaceutical industry, however, aloe is used primarily to make topical products such as gel preparations and ointments and is also incorporated into many capsules and tablets for oral use. In the food industry, gel and juice are used in a variety of beverages and other food products, especially in health food drinks formulations [9].

It is important to note that both *A. arborescens* and *A. barbadensis* increase their popularity by demonstrating many medicinal properties. They are credited with laxative, anti-inflammatory, immunostimulatory, antiseptic, antimicrobial, antidiabetic, and antitumor properties as well as helping to improve the healing of wounds and burns [11,12,13,14,15,16]. Due to the presence of anthraquinones (e.g., aloin, aloe-emodin, and chrysophanic acid) and their structures, aloes have cytotoxic properties and can damage pathogenic cells and therefore have antimicrobial effects [17,18,19,20,21].

Previous studies are mostly focused on the antimicrobial effect of *A. barbadensis* [22,23,24], while the study for antimicrobial effect of *A. arborescens* is significantly less detectable in the literature. Most studies cover the antimicrobial activity of aloe extracts [17,23,25,26,27,28,29], not fresh aloe samples. For this reason, the purpose of our comparative study was to examine the antimicrobial efficacy of fresh juice and gel of both *A. barbadensis* and *A. arborescens* and compare it with the antimicrobial efficacy of some aloe commercial products. Antimicrobial activity was tested against fungi (*Candida albicans*), Gram-negative (*Escherichia coli, Pseudomonas aeruginosa, Pseudomonas fluorescens*) and Gram-positive (*Bacillus cereus, Staphylococcus aureus*) bacteria with the aim to provide a natural source for antimicrobial treatments.

## 2. Results

### 2.1. Antimicrobial Efficacy of Aloe Samples on Fungi

The antimicrobial activity of natural and commercial aloe products on fungi was determined at two initial concentrations of *C. albicans*. Only commercial Aloe vera ESI^®^ gel and Fruit of the Earth gel^®^ showed antifungal activity. No inhibition zone was detected with the remaining samples (Table 1). As expected, the diameter of the inhibition zone decreased with higher initial concentration of yeast.

Since fresh samples did not show antimicrobial efficacy by the diffusion method, broth microdilution method was used for more accurate and quantitative antifungal efficacy at different concentrations of natural and commercial aloe products.

**Figure 1:**
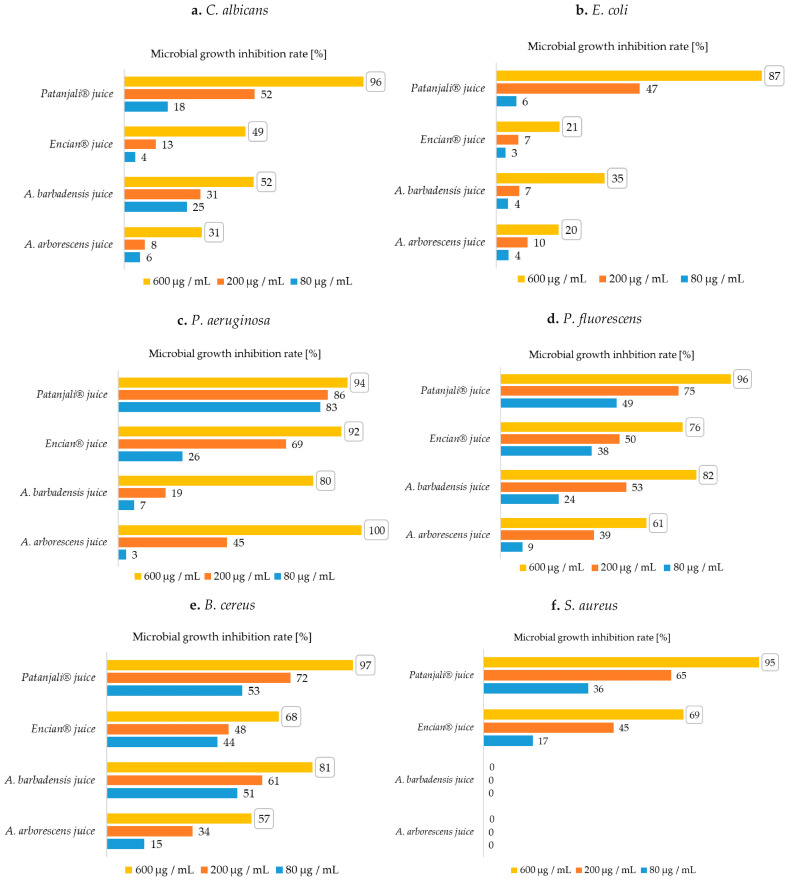
Microbial growth inhibition rates (MGIRs) for aloe juices using 600, 200, and 80 μg juice/mL suspension; (**a**)—MGIRs for *C. albicans*; (**b**)—MGIRs for *E. coli*, (**c**)—MGIRs for *P. aeruginosa*, (**d**)—MGIRs for *P. fluorescens*, (**e**)—MGIRs for *B. cereus*, (**f**)—MGIRs for *S. aureus*; The numbers in the boxes are the highest achieved MGIRs for each sample. (Initial concentrations of microorganisms: *C. albicans* 10^6^ CFU/mL, *E. coli* 10^7^ CFU/mL, *P. aeruginosa* 10^7^ CFU/mL, *P. fluorescens* 10^7^ CFU/mL, *B. cereus* 10^7^ CFU/mL, *S. aureus* 10^5^ CFU/mL); standard deviations were max. ± 3).

A comparison of the antimicrobial efficacy (quantitative determination of the microbial growth inhibition) of aloe juices on the growth of *C. albicans* is shown in Figure 1a.

According to the literature review, there are no studies covering quantitative testing of aloe juices for the growth of the fungus *C. albicans*. However, Alemdar and Agaoglu [30] showed that *A. barbadensis* juice has antimicrobial activity against *C. albicans*, while in another study, Jia et al. [8] found that *C. albicans* was not susceptible to the addition of *A. arborescens* juice. In our study, aloe juice was less effective in inhibiting the growth of *C. albicans*. Only Patanjali^®^ juice proved to be a good inhibitor, as it showed 97% microbial growth inhibition rate (MGIR) in the case of the highest added concentration (600 μg/mL), which in our case is also the minimum inhibitory concentration (MIC_90_) value. In the case of fresh aloe juices, *A. barbadensis* juice proved to be a better growth inhibitor, as it inhibited the growth of *C. albicans* microbial cells by 52% MGIR, while *A. arborescens* juice showed 31% inhibition of *C. albicans* growth. The results are consistent with the beforementioned literature data; as in the case of juices, *A. barbadensis* is a better growth inhibitor of yeast *C. albicans*.

**Figure 2:**
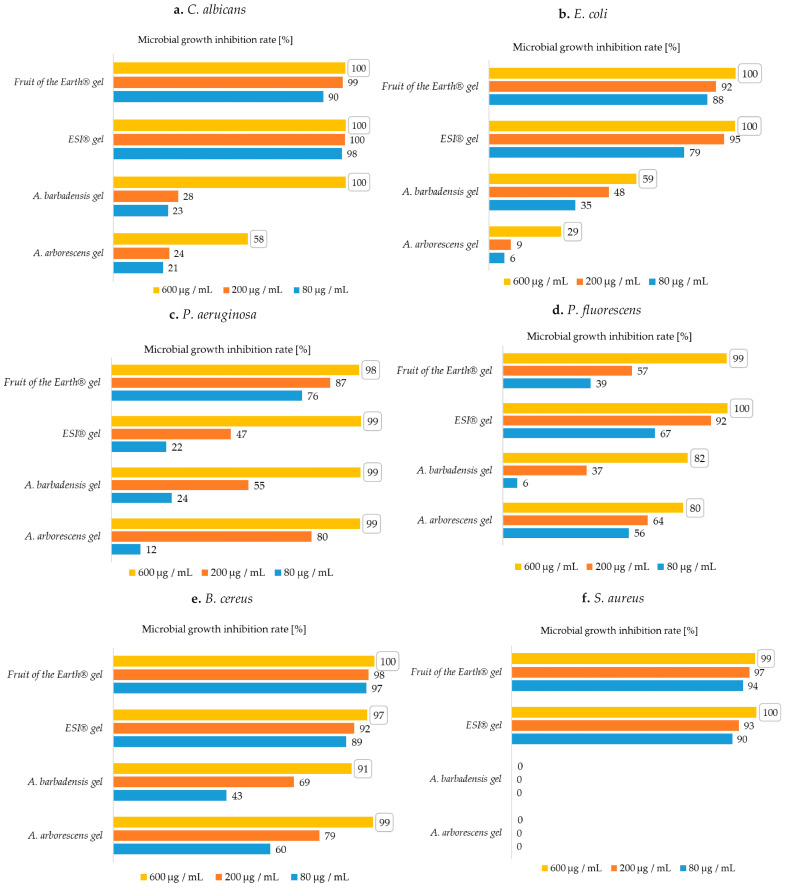
MGIRs for aloe gels using 600, 200, and 80 μg juice/mL suspension; (**a**)—MGIRs for *C. albicans*; (**b**)—MGIRs for *E. coli*, (**c**)—MGIRs for *P. aeruginosa*, (**d**)—MGIRs for *P. fluorescens*, (**e**)—MGIRs for *B. cereus*, (**f**)—MGIRs for *S. aureus*; The numbers in the boxes are the highest achieved MGIRs for each sample. (Initial concentrations of microorganisms: *C. albicans* 10^6^ CFU/mL, *E. coli* 10^7^ CFU/mL, *P. aeruginosa* 10^7^ CFU/mL, *P. fluorescens* 10^7^ CFU/mL, *B. cereus* 10^7^ CFU/mL, *S. aureus* 10^5^ CFU/mL); standard deviations were max. ± 3).

A comparison of the antimicrobial efficacy (quantitative determination of the microbial growth inhibition) of aloe gels on the growth of *C. albicans* is shown in Figure 2a.

Fruit of the Earth^®^ gel, ESI^®^ gel, and *A. barbadensis* gel have shown exceptional ability to inhibit the growth of *C. albicans*. All these samples achieved 100% MGIR at the highest tested concentration. Both commercial products perfectly inhibited *C. albicans* growth, even at the concentrations of 200 and 80 μg/mL, as they achieved MGIR greater than or equal to 90%. Thus, the MIC_90_ for both commercial gels could be determined as a concentration of 80 μg/mL. For a more accurate determination of the MIC value, additional research should be done, however, we determined a much lower MIC value than in the comparable study (50,000 μg/mL) [31]. The differences in determined MIC values could be due to different methodological procedures for sample preparation. The samples in this study were prepared at room temperature, while in the aforementioned study, the samples were treated at 85–90 °C. Such heating could lead to the destruction of the active components in the material, which in turn could lead to a lower degree of inhibition in the presence of the added sample [32]. Most likely, their heat treatment affected the activity of the active ingredients. Furthermore, the *A. arborescens* gel, which inhibited the growth of *C. albicans* with 58% MGIR at the highest added concentration, showed very poor inhibition properties.

### 2.2. Antimicrobial Efficacy of Aloe Samples on Gram-Negative Bacteria

Qualitative assessment of antimicrobial activity was performed at two concentrations of each bacteria. Table 2 shows the diameters of the inhibition zones.

In the case of Gram-negative bacteria, the disk diffusion method showed the inhibitory properties of Aloe vera ESI^®^ gel and Fruit of the Earth^®^ gel among all samples. Again, the diameter of the inhibition zone decreased with higher initial concentration of microbial culture. No inhibition zone was detected with fresh *A. arborescens* and *A. barbadensis* juice and gel.

A comparable study from Kaithwas et al. [33] with commercial products showed that commercial aloe vera gel showed a 6 mm inhibition zone in the cases of *E. coli* and *P. aeruginosa*, but the initial concentrations of microorganisms are unknown. In our study, both commercial gels displayed stronger inhibition, as the zones of inhibitions were equal to or greater than 14 mm. 

For more detailed results of antimicrobial activity of aloe samples on Gram-negative bacteria, the broth microdilution method was used.

A comparison of the antimicrobial efficacy of aloe juices on the growth of *E. coli* is shown in Figure 1b.

In terms of *E. coli* growth inhibition, Patanjali^®^ juice showed the highest values, achieving 87% MGIR at a concentration of 600 μg/mL. All other samples inhibited the growth of *E. coli* poorly. No references with specific MIC values or quantification of the antimicrobial efficacy of aloe juice on *E. coli* growth were detected. The antimicrobial activity of *A. arborescens* juice against *E. coli* was determined by monitoring the turbidity after incubation of microorganism [8].

A comparison of the antimicrobial efficacy of aloe gels on the growth of *E. coli* is shown in Figure 2b.

Despite the fact that aloe juices poorly inhibited the growth of *E. coli*, commercial gels in particular proved to be excellent inhibitors of the aforementioned microbial culture. At the highest added concentration (600 μg/mL), both commercial gels completely inhibited the growth of Gram-negative *E. coli* and further showed high MGIR at an added concentration of 200 μg/mL, which was also the MIC_90_ value for Fruit of the Earth^®^ and ESI^®^ gels. At a concentration of 80 μg/mL, Fruit of the Earth^®^ gel achieved 88% MGIR and ESI^®^ gel achieved 79% MGIR. As with fresh juices, *A. barbadensis* gel showed better inhibition of *E. coli* growth than *A. arborescens*. Cataldi et al. [31] determined the MIC for *A. barbadensis* gel at 400,000 μg/mL. Nevertheless, in the present research, a concentration of 600 μg/mL slightly inhibited the growth of *E. coli*, and with somewhat higher added concentration, we could probably also determine a much lower MIC value than in the previously mentioned study. Further studies are needed to determine the exact MIC value.

A comparison of the antimicrobial efficacy of aloe juices on the growth of *P. aeruginosa* is shown in Figure 1c.

Interestingly, *A. arborescens* juice inhibited *P. aeruginosa* growth completely at 600 μg/mL, followed by commercial Patanjali^®^ juice with 94% MGIR. Patanjali^®^ juice, however, is a better inhibitor; at lower concentrations (200 and 80 μg/mL), it achieved much higher rates of growth inhibition (86% and 83% MGIR). Encian^®^ juice inhibited the growth of *P. aeruginosa* with 92% MGIR. MIC_90_ values for both commercial products and *A. arborescens* juice were 600 μg/mL. At the same concentration, *A. barbadensis* juice showed 80% MGIR. The results are comparable to previously published studies showing that *P. aeruginosa* is sensitive to the addition of *A. arobrescens* juice [8]. While our study showed good inhibition of *P. aeruginosa* growth with the addition of *A. barbadensis* juice, Alemdar and Agaoglu [30] did not detect its antimicrobial efficacy against *P. aeruginosa*. Other quantitative results, such as MGIR or MIC values, are not available to compare the obtained antimicrobial activity of juices to *P. aeruginosa* growth with literature.

However, all tested aloe gels showed excellent inhibition of *P. aeruginosa* growth, as they showed greater than or equal to 98% MGIR (Figure 2c).

For all gels, the MIC_90_ value in the case of *P. aeruginosa* was 600 μg/mL, which is well below the MIC concentration of 400,000 μg/mL in another study [31]. Fruit of the Earth^®^ gel and *A. arborescens* gel stood out, reaching 87% and 80% MGIR at a concentration of 200 μg/mL. In the case of both gel and juice, *A. arborescens* better inhibited the growth of Gram-negative *P. aeruginosa*.

Among Gram-negative bacteria, we also defined the quantitative degree of antibacterial activity on the growth of *P. fluorescens*. A comparison of the antimicrobial efficacy of aloe juices on the growth of *P. fluorescens* is shown in Figure 1d.

In our study, Patanjali^®^ juice showed, among the tested juices, the best antimicrobial efficacy on *P. fluorescens* growth, and its MIC_90_ was detected at 600 μg/mL. However, some growth inhibition of *P. fluorescens* was also shown with the addition of 80 μg/mL. Among other juices, the highest inhibition was achieved by *A. barbadensis* juice, followed by Encian^®^ juice and *A. arborescens* juice at an added concentration of 600 μg/mL.

Furthermore, the gels also inhibited the growth of *P. fluorescens* (Figure 2d) even better than the juices.

Again, the commercial gels showed better antimicrobial activity. The best was ESI^®^ gel, which completely inhibited the growth of *P. fluorescens* at 600 μg/mL and at 200 μg/mL with 100% and 92% MGIR, respectively. In the case of using ESI^®^ gel, the MIC_90_ value was defined at 200 μg/mL, while in the case of using Fruit of the Earth^®^ gel as an MIC_90_ inhibitor, the value was defined at 600 μg/mL. Fresh gels inhibited growth of *P. fluorescens* with 80% and 82% MGIR at the highest added concentration. According to the results, *A. barbadensis* showed slightly higher antimicrobial efficacy on *P. fluorescens* than *A. arborescens*. As far as we know, no comparable studies containing antimicrobial testing on *P. fluorescens* with aloe juices and gels were published.

### 2.3. Antimicrobial Efficacy of Aloe Samples on Gram-Positive Bacteria

The antimicrobial efficacy of natural and commercial samples of aloe on Gram-positive bacteria was qualitatively determined at two initial concentrations for each microorganism. Table 3 shows diameters of inhibition zones for Gram-positive bacteria.

In the case of Gram-positive bacteria, the disk diffusion method showed the inhibitory properties of only Aloe vera ESI^®^ and Fruit of the Earth^®^ gel among all samples. Both commercial products inhibited growth of *B. cereus*, while only Fruit of the Earth^®^ gel inhibited growth of *S. aureus*. Results of our study showed a 21–25 mm inhibition zone for Fruit of the Earth^®^ gel, while Kaithwas et al. [33] determined a 10 mm zone of inhibition in the case of *S. aureus*, where initial concentration of microbial culture is not known. As expected, the diameter of the inhibition zone decreased with higher initial concentration of microorganisms. No inhibition zone was detected at fresh *A. arborescens* and *A. barbadensis* juice and gel.

Further, the broth microdilution method was used to determine the MGIR of Gram-positive bacteria at different concentrations of the added samples.

A comparison of the antimicrobial efficacy of aloe juices on the growth of *B. cereus* is shown in Figure 1e.

In comparison with juices as inhibitors, the commercial product Patanjali^®^ juice proved to be the best at inhibiting growth of *B. cereus* at an added concentration of 600 μg/mL. *A. barbadensis* juice with the highest added concentration inhibited the growth of *B. cereus* with 81% MGIR, which ranks it immediately after the inhibitory effect of Patanjali^®^ juice. They are followed by Encian^®^ juice and *A. arborescens* juice. Among commercial juices, Patanjali^®^ juice inhibited the growth of *B. cereus* most effectively. According to the MGIR of Patanjali^®^ juice, the MIC_90_ value could be determined at a concentration of 600 μg/mL in our case. For more accurate determination, antimicrobial activity should be tested at concentrations between 200 and 600 μg/mL. On the other hand, among fresh samples, *A. barbadensis* juice was more successful in inhibiting growth of *B. cereus*. 

A comparison of the antimicrobial efficacy of aloe gels on the growth of *B. cereus* is shown in Figure 2e. 

Furthermore, all tested aloe gels showed exceptionally good growth inhibition of Gram-positive bacterium *B. cereus*, as all samples achieved MGIR above 90% at a concentration of 600 μg/mL. Both commercial products have been successful in inhibiting growth of *B. cereus*. MIC_90_ value can be determined for both samples, namely for ESI^®^ gel at a concentration of 200 μg/mL and for Fruit of the Earth^®^ gel already at a concentration of 80 μg/mL. Fresh gels of *A. arborescens* and *A. barbadensis* are also excellent inhibitors of *B. cereus* growth. *A. barbadensis* gel achieved 91% MGIR, while *A. arborescens* gel achieved as much as 99% MGIR at a concentration of 600 μg/mL. At this concentration, the MIC_90_ value could also be determined for both fresh samples.

Moreover, antimicrobial activity of natural and commercial samples of *A. arborescens* and *A. barbadensis* on *S. aureus* growth was determined.

Jia et al. [8] state the antimicrobial efficacy of *A. arborescens* juice on *S. aureus* growth, while the results of the broth microdilution method showed that fresh aloe juices and gels (Figure 1f and Figure 2f) did not inhibit the growth of Gram-positive *S. aureus*. The results are comparable to a study by researchers Alemdar and Agaoglu [30], where aloe vera showed no antimicrobial activity against *S. aureus*. Furthermore, concentrations in our study immeasurably lower than those of Cataldi et al. [31] were found to inhibit the growth of *S. aureus*, while the MIC value was determined at 800,000 μg/mL.

Comparing the results for commercial products (Figure 2f), both gels were excellent growth inhibitors, reaching 90% (ESI^®^ gel) and 94% MGIR (Fruit of the Earth^®^ gel) at the lowest added concentration (80 μg/mL). Thus, the MIC_90_ value for both gels was defined as 80 μg/mL. Regarding the antimicrobial efficacy of commercial juices, the growth of *S. aureus* was best inhibited by Patanjali^®^ juice. This inhibited the growth of *S. aureus* with MIC_90_ value at a concentration of 600 μg/mL. The weakest inhibitor was Encian^®^ juice, which showed 69% MGIR at a concentration of 600 μg/mL.

## 3. Discussion

The qualitative disk diffusion method has only detected inhibition by commercial gels (Fruit of the Earth^®^ gel and ESI^®^ gel). It should be noted that commercial products contain additional antimicrobial components, such as tea tree (*Melaleuca alternifolia*) oil [34] in the case of ESI^®^ gel, which could contribute to a better inhibitory effect through a synergistic effect. Antimicrobial additives and preservatives used in commercial products are also triethanolamine [35], DMDM hydantoin [36], diazolidinyl urea, [37], EDTA [38], tocopheryl acetate [39], phenoxyethanol [40], potassium sorbate [41], linalool [42], limonene [43], citric acid [44], ascorbic acid [45], and sodium benzoate [46]. If two substances have similar effects, it is exploited to obtain the same effect with lower doses of each active substance [47].

As the results of the qualitative method were not adequate, the inhibitory properties of the samples on the growth of microbial cultures were tested by the quantitative broth microdilution method. 

Regarding commercial products as growth inhibitors of the tested microorganisms, both commercial gels proved to be the strongest. Fruit of the Earth^®^ gel inhibited the growth of *B. cereus* and *E. coli* among all commercial products the strongest, while ESI^®^ gel gave the strongest inhibition for the growth of *C. albicans*, *P. aeruginosa*, *P. fluorescens*, and *S. aureus*.

The results showed that *B. cereus* growth was the most inhibited by *A. arborescens* gel, considering natural samples of both aloes. Furthermore, looking at natural aloe samples, *A. barbadensis* gel was the strongest growth inhibitor for *C. albicans* and *E. coli* growth, while *A. arborescens* juice gave the larger inhibition zone for *P. aeruginosa* growth, and *A. barbadensis* juice was the strongest inhibitor in the case of *P. fluorescens* growth. Natural samples of both tested aloes did not inhibit the growth of Gram-positive culture *S. aureus*. Of particular interest is the fact that the highest concentrations of natural juices and gels without additives completely inhibit the growth of *B. cereus*, *C. albicans*, and *P. aeruginosa*. It should be noted that, at higher concentrations of samples, natural products are comparable to commercial ones, while at lower concentrations, commercial products achieve significantly more effective inhibition of the growth of microorganisms.

Because there is a lack of studies in the literature with completely natural samples of *A. barbadensis* and *A. arborescens*, and, above all, *A. arborescens* is even less well known, it is important to compare their results. Both *A. barbadensis* juice and gel inhibited the growth of *C. albicans*, *E. coli*, and *P. fluorescens* better than *A. arborescens*. In the case of *P. fluorescens* gel, MGIRs were almost the same when the highest concentration was added, while the lower concentration of *A. arborescens* gel better inhibited the growth of the aforementioned microorganism. The gels of both aloes inhibited the growth of *P. aeruginosa* at the highest added concentration at the same rate, while the gel of *A. arborescens* performed better at the added lower concentration (200 μg/mL). In the case of Gram-positive *B. cereus*, however, *A. barbadensis* juice was a better inhibitor than *A. arborescens* juice, whereas *A. arborescens* gel inhibited *B. cereus* growth better than *A. barbadensis* gel at all added concentrations.

## 4. Materials and Methods

### 4.1. Chemicals

Peptone from soybean, yeast extract, and agar were obtained from Sigma-Aldrich, St. Luis, MO, USA. Sodium chloride, ethanol, meat extract, and meat peptone were obtained from Merck, Darmstadt, Germany. Triptic soy broth, tryptone, potato dextrose broth, and malt extract were obtained from Fluka, Buchs, Switzerland. Potato dextrose agar was from Biolife, Milano, Italy, while anhydrous D-(+)-glucose was obtained from Kemika, Zagreb, Croatia.

### 4.2. Microorganisms

Bacterial and fungal strains were provided from DSMZ-German Collection of Microorganisms and Cell Cultures GmbH, Germany. Antimicrobial activity of samples was determined against different pathogenic microbes, including fungi (*Candida albicans* DSM 1386), Gram-negative (*Escherichia coli* DSM 498, *Pseudomonas aeruginosa* DSM 1128, *Pseudomonas fluorescens* DSM 289), and Gram-positive (*Bacillus cereus* DSM 345, *Staphylococcus aureus* DSM 346) bacteria.

### 4.3. Plant Material Preparation

Mature and fresh leaves of *A. arborescens* and *A. barbadensis*, approximately 50–80 cm long, were washed under water. Using a knife, the cores of *A. arborescens* and *A. barbadensis* were separated from the thick outer layer of the leaves. The inner cores were cut into pieces and centrifuged (Eppendorf Centrifuge 5840R, Deutschland) at 11,000 rpm, 23 °C for 15 min. The transparent juice was decanted. The transparent and mucilaginous gel was then collected and homogenized. Natural juices and gels were stored at 4 °C until further use.

### 4.4. Commercial Products

Aloe vera 100% gel from Fruit of the Earth^®^, Aloe vera ESI^®^ gel from ESI^®^, and Aloe vera Juice with pulp of Aloe vera from Encian^®^ were obtained from local health food stores. Aloe vera Juice from Patanjali^®^ was obtained from India. According to the label, Aloe vera 100% gel from Fruit of the Earth^®^ contains *A. barbadensis* leaf juice, triethanolamine, carbomer, DMDM hydantoin, diazolidinyl urea, tetrasodium EDTA, and tocopheryl acetate. Aloe vera ESI^®^ gel contains aqua, *A. barbadensis* leaf, acrylates/c10-30 alkyl acrylate crosspolymer, tocopheryl acetate (vitamin E), *Melaleuca alternifolia* leaf oil (tea tree), disodium EDTA, sodium hydroxide, phenoxyethanol, potassium sorbate, benzoic acid, linalool, and limonene. Aloe vera Juice from Encian^®^ contains *A. barbadensis* leaf juice, citric acid, and ascorbic acid (vitamin C), and the guaranteed minimum *A. barbadensis* content is 0.99 g/mL. Aloe vera Juice from Patanjali^®^ contains *A. barbadensis* leaf juice (0.95 mL/mL), sorbitol, citric acid, carrageenan, sodium benzoate, and potassium sorbate.

### 4.5. General Method for Qualitative Determination of Antimicrobial Activity of Samples

For qualitative determination of antimicrobial activity of aloe samples, a Kirby–Bauer disk diffusion method [48] was used. A total of 100 µL of microorganism solution was smeared on the agar plates. Then, 9 mm sterile cellulose discs were put on the inoculated agar plate. Further, 50 µL of potential antimicrobial sample was applied on the disc. Additionally, a control or a “blank” was applied, as 50 µL of physiological solution was placed on sterile cellulose disc. The incubation of the inoculated plates took place under optimal conditions, i.e., at the optimal temperature (Table 4) for each individual microorganism for 24 h. After the incubation period, the diameter of the resulting inhibition zone was measured, which was a scale for antimicrobial efficiency of the samples. All qualitative tests were performed in triplicates, and all associated standard deviations are shown separately in each subchapter.

### 4.6. General Method for Quantitative Determination of the Microbial Growth Inhibition Rate

For quantitative determination of antimicrobial activity, a broth microdilution method [49] was used. A sterile inoculation loop was used to transfer the microorganism to the prepared broth (9mL). The initial concentration (Table 5) was determined by spread-plate technique. Different amounts of the sample and the medium/suspension of the microorganism culture were pipetted into 96-well microtiter plates to achieve different concentrations (80, 200, or 600 μg of sample/mL of suspension) of potentially antimicrobial samples. Sterility control and growth control of microorganisms were also prepared. The optical density (OD) of the prepared dilutions was measured using a Tecan Infinite F200 spectrophotometer. OD was measured for 12 h under optimal conditions for the growth of each microorganism. Five seconds of stirring with an amplitude of 2 mm was applied prior to every read of OD, which were measured every 30 min for three hours and then every 60 min until the end of the experiment. Based on the obtained results, the microbial growth inhibition rate (MGIR) was calculated. MGIR was determined based on OD of the growth control and OD of the sample according to the following equations:

OD (control culture) = OD (suspension of the microorganism culture) − OD (broth)(1)

OD (sample) = OD (suspension of the microorganism culture with sample) − OD (broth with sample)(2)

Microbial growth inhibition rate (MGIR) (%) = ((OD (control culture) − OD (sample))/ OD (control culture)) × 100(3)

The minimum inhibitory concentration (MIC_90_) was determined as the concentration at which the sample inhibits the growth of the microorganism by at least 90% MGIR. All quantitative tests were performed in triplicates, and all associated standard deviations are shown separately in each subchapter.

## 5. Conclusions

Our research confirmed the antimicrobial effect of both aloe species, *A. arborescens* and *A. barbadensis*. All samples inhibited the growth of all microbial cells, except for fresh samples of aloes, which did not inhibit the growth of *S. aureus*. The most interesting are the results of the inhibition of the growth of *B. cereus*, *C. albicans*, and *P. aeruginosa* as fresh juices and gels, comparable to commercial products at high concentrations of samples added, which perfectly inhibited the growth of these microorganisms. *A. arborescens* gel showed the strongest inhibitory properties for the growth of Gram-positive *B. cereus*, the growth of fungi *C. albicans* was completely inhibited by *A. barbadensis* gel, and the growth of Gram-positive *P. aeruginosa* was completely inhibited by *A. arborescens* juice.

Results of this study are very important, as *B. cereus* is often present in raw, dried, or cooked food and can cause various food-borne diseases [50]. *C. albicans* can cause opportunistic infection and most commonly causes well-known candidiasis [51]. Additionally, last but not least, one of the most common infections is with *P. aeruginosa*, which often develops drug resistance [52].

It is important to point out that *A. barbadensis* is the main ingredient of all tested commercial products, to which many antimicrobial additives or preservatives are added, which, with a synergistic effect, enable even better antimicrobial efficacy. With our study, we confirmed the excellent antimicrobial effect of all-natural *A. barbadensis*. Moreover, the important conclusion of our study is the antimicrobial activity of the lesser-known *A. arborescens*, which shows great potential for use in various research areas and for further applications in cosmetic, food, and pharmaceutical industries.

Antimicrobial agents available in nature can make a significant contribution to new antimicrobial drugs by eliminating or reducing antimicrobial resistance.

## Figures and Tables

**Table 1:** Antimicrobial activity of aloes natural and commercial products on fungi.

Fungi	Diameter of the Inhibition Zone [mm]	
	ESI^®^ Gel	Fruit of the Earth^®^ Gel
*C. albicans*	17 ± 2 15 ± 1	18 ± 2 10 ± 1

All data are expressed as mean ± standard deviation.

**Table 2:** Antimicrobial activity of aloes natural and commercial products on Gram-negative bacteria.

Gram-Negative Bacteria	Diameter of the Inhibition Zone (mm)	
	ESI^®^ Gel	Fruit of the Earth^®^ Gel
*E. coli*	14 ± 2	32 ± 2
	-	30 ± 2
*P. aeruginosa*	35 ± 3	31 ± 2
	30 ± 2	24 ± 2
*P. fluorescens*	20 ± 2	45 ± 3
	14 ± 1	32 ± 2

All data are expressed as mean ± standard deviation.

**Table 3:** Antimicrobial activity of aloes natural and commercial products on Gram-positive bacteria.

Gram-Positive Bacteria	Diameter of the Inhibition Zone [mm]	
	ESI^®^ Gel	Fruit of the Earth^®^ Gel
*B. cereus*	20 ± 2	33 ± 3
	17 ± 1	23 ± 3
*S. aureus*	-	25 ± 2
	-	21 ± 2

All data are expressed as mean ± standard deviation.

**Table 4:** Optimal conditions and initial concentrations of microorganisms for qualitative determination of antimicrobial activity.

Microorganism	Optimal Temperature	Initial Concentrations
*C. albicans*	25 °C	10^5^ CFU/mL 10^7^ CFU/mL
*E. coli*	37 °C	10^6^ CFU/mL 10^7^ CFU/mL
*P. aeruginosa*	37 °C	10^6^ CFU/mL 10^8^ CFU/ml
*P. fluorescens*	30 °C	10^6^ CFU/mL 10^7^ CFU/mL
*B. cereus*	30 °C	10^6^ CFU/mL 10^7^ CFU/mL
*S. aureus*	37 °C	10^5^ CFU/mL 10^8^ CFU/mL

**Table 5:** Optimal conditions and initial concentrations of microorganisms for quantitative determination of antimicrobial activity.

Microorganism	Optimal Temperature	Initial Concentrations
*C. albicans*	25 °C	10^6^ CFU/mL
*E. coli*	37 °C	10^7^ CFU/mL
*P. aeruginosa*	37 °C	10^7^ CFU/mL
*P. fluorescens*	30 °C	10^7^ CFU/mL
*B. cereus*	30 °C	10^7^ CFU/mL
*S. aureus*	37 °C	10^5^ CFU/mL
